# Left atrial endocarditis as a rare complication of mitral valve endocarditis: a clinical case

**DOI:** 10.1186/1471-2261-12-103

**Published:** 2012-11-15

**Authors:** Ali Hamadanchi, Wolfgang Bothe, Alexander Pfeil, Azita Abdi Rad, Bernhard R Brehm, Hans R Figulla, Thorsten Doenst, Marcus Franz, Christian Jung

**Affiliations:** 1 Department of Cardiology, University Hospital Jena, Erlanger Allee 101, D-07747, Jena, Germany; 2 Department of Cardiothoracic Surgery, University Hospital Jena, Jena, Germany; 3 Department of Internal medicine III, University Hospital Jena, Jena, Germany; 4 Institute of Pathology, University Hospital Jena, Jena, Germany

## Abstract

**Background:**

Infective Endocarditis (IE) is considered as a multifaceted problem in every aspect from etiology and presentation to diagnosis and management. Early recognition of this disease and especially its complications, remain a critical task for the cardiologist. Atrial endocarditis is a rare and sometimes unrecognized complication of mitral valve endocarditis.

**Case presentation:**

We present a 48 year-old male patient who was admitted to our clinic because of recent onset of malaise, fever, jaundice and peripheral edema. Important physical findings were peripheral stigmata of IE in addition to holosystolic murmur over the left sternal border. Transthoracic and transesophageal echocardiophy revealed a severe eccentric MR due to a flailed posterior mitral valve caused by IE. The presence of atrial septal endocarditis caused by jet streaming was also observed. Blood culture was positive for *streptococcus oralis* and antibiotic therapy was immediately initiated. Considering the large burden of infective tissue, the patient was planned for an early surgical intervention. A minimally invasive resection of the atrial mass, direct closure of the defect, resection of the diseased portions of mitral leaflets and implantation of a biological mitral valve prosthesis was performed. Intra-operative and histological findings confirmed provisional diagnosis by echocardiography.

**Conclusions:**

Together with comprehensive echocardiographic evaluation, attention should be placed on mural vegetations and excluded among all cases of mitral valve endocarditis, particularly those with severe eccentric regurgitant jets.

## Background

The clinical manifestations of endocarditis are very diverse. Although heart valves are the most common site of involvement, vegetations may also occur in other intracardiac locations. Echocardiography plays a pivotal role in the diagnosis of this disease and its complications.

Since the spread of pathogens is dependent on the blood stream, most complications occur distal to the source of infection. However, atrial endocarditis (AE) due to mitral valve endocarditis (MVE) is a rare complication that occurs more proximally, which can be explained by two major mechanisms: the “jet stream effect” (jet lesions) and “direct spreading” of infection from the adjacent infected tissues [1,2]. Another possible etiology of an endocarditis of the atrial septum is recognized to occur following percutaneous closure of atrial septal defects.

No data is currently available that describes the prevalence or clinical impact of AE. Whether this complication is truly rare or underestimated still remains to be known. To address this deficit, we describe a case of AE due to associated MVE that resulted in poor outcome.

## Case presentation

A 48-year old man with a history of mild asymptomatic mitral regurgitation (MR) was admitted to our hospital because of recent onset of malaise, fever, jaundice and peripheral edema. The patient was a known case of alcoholic liver disease (Child A). He denied any history of nausea, vomiting, gastrointestinal bleeding, dental extraction, or recent weight loss. On examination, he had a temperature of 39°C, a pulse rate of 120 beats/min and blood pressure of 106/70 mmHg. Other notable findings were the presence of poor dental hygiene, a loud grade 4/6 holosystolic murmur (best heard over left sternal border with thrill and radiation to the right sternal border), Osler’s nodes in the palms, and lower limb edema. Routine laboratory exams showed mildly elevated liver function tests (ASAT 2.86 μmol/l, ALAT 1.71 μmol/l), leukocytosis (17.8 Gpt/l), elevated CRP (98.0 mg/l) and normal renal function tests. Electrocardiography was unremarkable.

**Figure 1:**
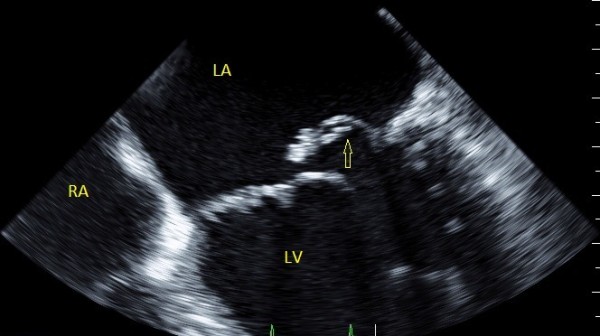
TEE showing the flail Posterior Mitral Leaflet (P2), with associated vegetations and a large regurgitant orifice area.

**Figure 2:**
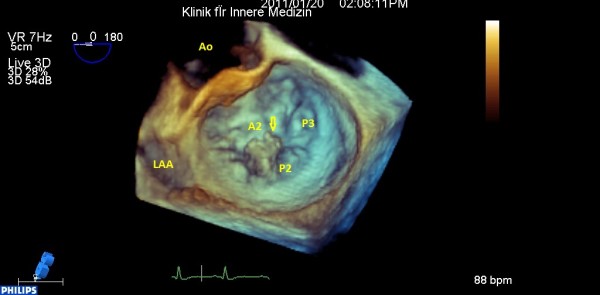
**Three dimensional surgical view of MV, flail P**_**2**_**and small vegetations.**

**Figure 3:**
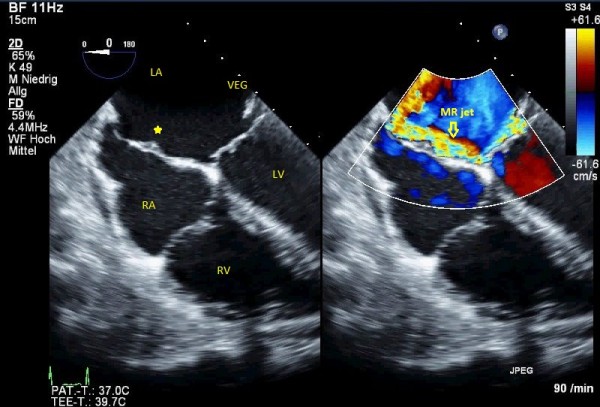
Bi-caval view in TEE, small aneurysm of IAS is filled a homogenouse sessile mass consistent with jet lesion vegetation (left) and color compare mode to show the eccentric jet of MR and jet lesions on IAS, arrowhead indicates the atrial vegetation (right).

**Figure 4:**
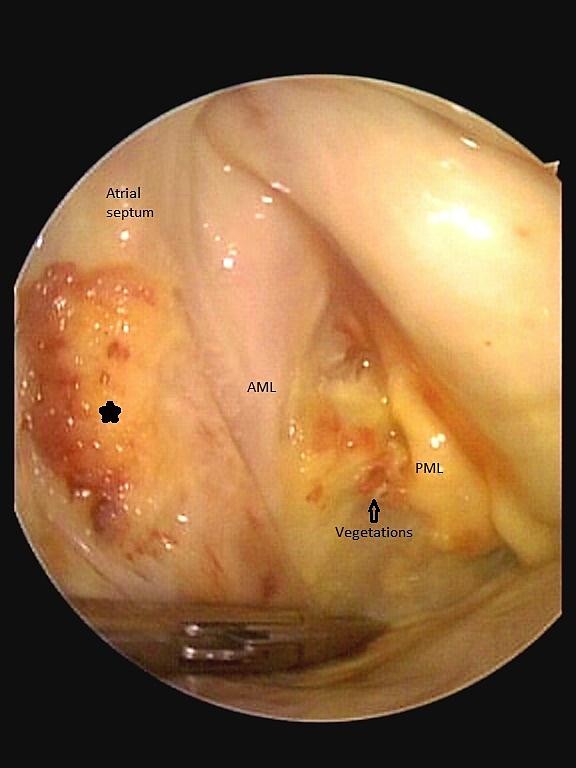
The intraoperative findings with correlated reconstructed 3D TEE image, the asterisk indicate the atrial vegetation, the “footprints” of MV endocarditis.

**Figure 5:**
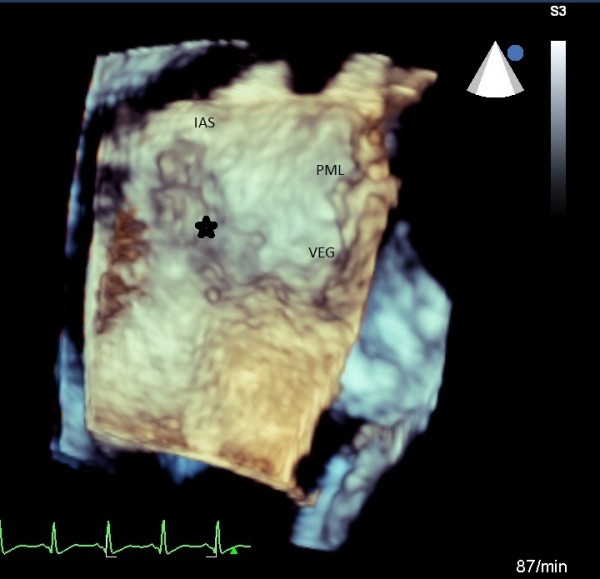
The intraoperative findings with correlated reconstructed 3D TEE image, the asterisk indicate the atrial vegetation, the “footprints” of MV endocarditis.

**Figure 6:**
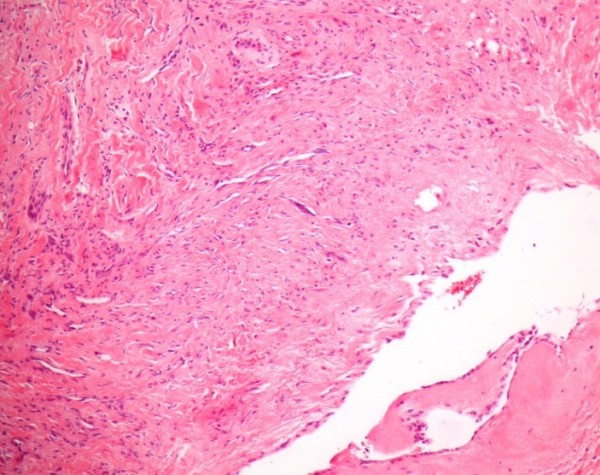
Histological findings from the resected atrial septum showing an old focal subendocardial bleeding, a focal mild to moderate fibrosis and some neutrophil granulocytes.

Transthoracic echocardiography (TTE) revealed a flail posterior mitral leaflet (P_2_) with multiple flickering vegetations, and severe centrally originating eccentric jet of mitral regurgitation towards the atrial septum (medially and anteriorly). Following an upper gastrointestinal endoscopy, which revealed Grade I of lower esophageal varices, tranesophageal echocardiophy (TEE) was performed the following day, confirming TTE findings (Figures 1, 2 and 3). It is worth noting that on close scanning of the aneurysmal interatrial septum (IAS) an overlying relatively fixed and sessile echogenic mass (30 × 10 mm) attached was observed. Although thrombus formation was a differential diagnosis, our first provisional diagnosis was AE, keeping effects of jet lesions in mind (Figures 2 and 3; Additional file 1: Movie 1 and Additional file 2: Movie 2). Three Dimensional TEE (3D-TEE) facilitated us in delineation of the lesion. The blood culture was found to be positive for *Streptococcus oralis* and treatment was started immediately with penicillin G and other supportive medications. After 7 days of antibiotic therapy, although hemodynamically stable, a consensus decision (following discussion with patient) was made for early surgical intervention. Decision for surgical intervention was based on the large burden of infective tissue on the Mitral Valve (MV) and IAS. Using cardiopulmonary bypass (CPB) and cardioplegic arrest, the patient underwent minimally invasive resection of the atrial mass (Figures 4 and 5), direct closure of the defect, resection of the diseased portions of anterior (surgical finding) and posterior mitral leaflet and implantation of a biological mitral valve prosthesis (Epic™ stented tissue valve, St. Jude Medical # 33). The patient was weaned from CPB and admitted to the intensive care unit. Culture of the specimens was negative for bacterial growth. Histopathological evaluation of resected tissues from mitral valve and atrial septum (Figure 6) was conclusive for atrial and mitral valve endocarditis, showing an old focal subendocardial bleeding, a focal mild to moderate fibrosis and presence of some neutrophil granulocytes.

During the postoperative course, the patient developed a secondary intracranial hemorrhage with right hemiplegia, likely explained as a result of endocarditic micro-embolization and late hemorrhagic transformation. The bleeding was drained surgically and the patient was sent to rehabilitation. It was reported that he died several weeks later because of sudden cardiopulmonary arrest. Autopsy permission was denied by patient’s relatives.

## Conclusions

Infective Endocarditis is usually considered as a multifaceted problem, from etiology and presentation to diagnosis and management. Early recognition of the disease and its complications remain a critical task for all physicians. In this era, the role of echocardiography, particularly TEE, is literally irreplaceable. It should be noted that in our case, initial TTE failed to locate the atrial septal lesions, and subsequent 2D/3D TEE helped to provide better delineation of the pathologies.

Our case demonstrates an illustrative example of AE as a result of a jet lesion. We propose that in cases of AE associated with MVE, which could be overlooked in routine clinical practice, the actual burden of vegetations is possibly greater than MVE alone, and therefore, recognition of AE may have important implications for the indication and timing of surgery among patients with MVE. Why every MVE with regurgitation is not complicated by AE or atrial septal endocarditis remains unclear, however the velocity and volume of regurgitant jet, the infective burden of the MV vegetations, the virulence of bacteria, host immuno-competence and finally the size of left atrium might play a role.

In conclusion, together with comprehensive echocardiographic evaluation, mural vegetations and AE should be considered and meticulously excluded among all cases of MVE, particularly those with high velocity eccentric regurgitant jets. For better eradication of the lesions, the possibility of AE should specifically be reported to the surgeon. Since a diagnostic TTE is rare in such circumstances, (3D-) TEE might be the optimal diagnostic means for optimal evaluation of the lesions and associated complications and can only be omitted if TTE is conclusive. Nevertheless, accurate surgical inspection is especially warranted in all eccentric jets for evaluation of jet lesions.

## Consent

The patient provided written informed consent for the publication of this report and for any accompanying images and videos.

## Abbreviations

LA: Left atrium; LV: Left Ventricle; RA: Right Atrium; IAS: Interatrial septum; AML: Anterior Mitral Leaflet; PML: Posterior Mitral Leaflet; VEG: Vegetation; SVC: Superior Vena Cava.

## Competing interests

The authors declare that they have no competing interests.

## Authors’ contributions

AH, WB, AP, AAR, BRB, HRF, TD, MF and CJ were all responsible for the treatment of the patient. AH, AP, BRB, HRF, MF and CJ performed echocardiographic evaluations. WB and TD operated on the patient and took intraoperative images. AAR and MF acquired the histological findings. AH wrote the first draft of the manuscript. All other authors revised the manuscript. All authors gave their final approval for the submission of the manuscript.

## Pre-publication history

The pre-publication history for this paper can be accessed here:

http://www.biomedcentral.com/1471-2261/12/103/prepub

## Supplementary Material

Additional file 1 **Movie 1.** TEE showing the flail Posterior Mitral Leaflet (P2), with associated vegetations and a large regurgitant orifice area. Click here for file

Additional file 2 **Movie 2.** Three dimensional surgical view of MV, flail P_2_ and small vegetations. Click here for file
